# New "light" for one-world approach toward safe and effective control of animal diseases and insect vectors from leishmaniac perspectives

**DOI:** 10.1186/s13071-016-1674-3

**Published:** 2016-07-13

**Authors:** Kwang Poo Chang, Bala K. Kolli, the New Light Group, Ramesh B. Batchu, Hui-Wen Chen, Larry Ming C. Chow, Robert Elliott, Jonathan F. Head, Chia-Kwung Fan, Chen-Hsiung Hung, Dar-Der Ji, Zhao-Rong Lun, Laura Manna, Yoshitsugu Matsumoto, Dennis K. P. Ng, Camila I. de Oliveira, Sayonara Melo, Yusuf Ozbel, Ahmet Özbilgin, Joseph Reynolds, Chizu Sanjoba, Shin-Hong Shiao, Nang-Yao Shih, Chi-Wei Tsai, Maria da Graça H. Vicente, Charles H. Barré, Petr Volf, Yueh-Lung Wu, Chao-Lan Yu, Xiao-Nong Zhou

**Affiliations:** Department of Microbiology/Immunology, Chicago Medical School/Rosalind Franklin University of Medicine and Science, 3333 Green Bay Rd, North Chicago, IL 60064 USA

**Keywords:** Photosensitizers, *Leishmania*, Mosquito, Photodynamic therapy, Photodynamic vaccination, Photodynamic insecticide

## Abstract

Light is known to excite photosensitizers (PS) to produce cytotoxic reactive oxygen species (ROS) in the presence of oxygen. This modality is attractive for designing control measures against animal diseases and pests. Many PS have a proven safety record. Also, the ROS cytotoxicity selects no resistant mutants, unlike other drugs and pesticides. Photodynamic therapy (PDT) refers to the use of PS as light activable tumoricides, microbicides and pesticides in medicine and agriculture.

Here we describe “photodynamic vaccination” (PDV) that uses PDT-inactivation of parasites, i.e. *Leishmania* as whole-cell vaccines against leishmaniasis, and as a universal carrier to deliver transgenic add-on vaccines against other infectious and malignant diseases. The efficacy of *Leishmania* for vaccine delivery makes use of their inherent attributes to parasitize antigen (vaccine)-presenting cells. Inactivation of *Leishmania* by PDT provides safety for their use. This is accomplished in two different ways: (i) chemical engineering of PS to enhance their uptake, e.g. Si-phthalocyanines; and (ii) transgenic approach to render *Leishmania* inducible for porphyrinogenesis. Three different schemes of *Leishmania*-based PDV are presented diagrammatically to depict the cellular events resulting in cell-mediated immunity, as seen experimentally against leishmaniasis and *Leishmania*-delivered antigen in vitro and in vivo. Safety *versus* efficacy evaluations are under way for PDT-inactivated *Leishmania*, including those further processed to facilitate their storage and transport. *Leishmania* transfected to express cancer and viral vaccine candidates are being prepared accordingly for experimental trials.

We have begun to examine PS-mediated photodynamic insecticides (PDI). Mosquito cells take up rose bengal/cyanosine, rendering them light-sensitive to undergo disintegration in vitro, thereby providing a cellular basis for the larvicidal activity seen by the same treatments. Ineffectiveness of phthalocyanines and porphyrins for PDI underscores its requirement for different PS. Differential uptake of PS by insect *versus* other cells to account for this difference is under study.

The ongoing work is patterned after the one-world approach by enlisting the participation of experts in medicinal chemistry, cell/molecular biology, immunology, parasitology, entomology, cancer research, tropical medicine and veterinary medicine. The availability of multidisciplinary expertise is indispensable for implementation of the necessary studies to move the project toward product development.

## Background

### Photosensitizers (PS)

These are ring compounds whose soluble form is light-excitable to produce cytotoxic reactive oxygen species (ROS) [1]. Naturally occurring PS include tetrapyrroles, e.g. corrins, chlorins and porphyrins – intermediates in the biosynthesis of vitamin B12, chlorophyll and heme [2]. The stoichiometry of these intermediates is stringently regulated by necessity to minimize their phototoxicity. Many plants produce PS as secondary metabolites for self-protection, e.g. psoralen and hypericin [3]. Other PS are chemically synthesized: the fluorescein analogues, rose bengal and cyanosine, and phthalocyanines (PC). Natural and synthetic PS include Food and Drug Administration (FDA)-approved drugs, cosmetic, food and fabric dyes.

### PDT-generated singlet oxygen (^1^O_2_) and -cell susceptibility

PDT has been used to eliminate tumors, pathogens and pests with cytotoxic ROS that is produced by illumination of targets treated with PS, e.g. porphyrins, PC and rose bengal, at their respective excitation wavelengths [4]. PDT initially generates singlet oxygen (^1^O_2_) and/or hydroxyl radicals, leading to the production of additional ROS, including peroxides and superoxides. ^1^O_2_ is highly reactive and destructive, but too short-lived (2–3 μs) to cross the cell membrane. ^1^O_2_ is produced by plants during photosynthesis, but not by non-photosynthetic mammals, insects and *Leishmania*. Cells from the latter group are thus most susceptible to oxidative damage by ^1^O_2_ because they lack mechanisms of detoxification. ^1^O_2_ has the potential for strategic deployment to inflict maximal destruction of specific cell types with minimal collateral damage.

PDT, especially using ^1^O_2_ generating PS for non-photosynthetic cells, is unlikely to select for resistance, since neither light nor PS alone is cytotoxic. Their use in combination produces ROS inactivating multiple targets, minimizing the likelihood of selecting resistant traits. In support of this concept, no resistant *Leishmania* were selected after six consecutive cycles of PDT, i.e. induced uroporphyrinogensis plus light (see below) [5, 6]. Few survivors emerged after each PDT cycle as aporphyric cells, resulting from reduced uptake of the inducer and/or heightened efflux of uroporphyrin I (URO). These phenotypes are not stable traits, since populations from the survivors after each of the six PDT cycles remain equally sensitive to the same PDT. Total inactivation of *Leishmania* by PDT is achievable when using two different PS, i.e. URO and PC (see below).

### Cellular uptake and subcellular targeting of PS for effective PDT

The effectiveness of PDT is a function of light intensity delivered at a wavelength specific to the PS and its quantum yield [4]. Under physiological conditions, PDT is critically dependent on the uptake of PS by the target cells. The best example to illustrate this is the all-or-none phototoxicity of the ^1^O_2_ generating URO, depending on its presence in the cytosol or in the extracellular milieu [5, 6]. URO is highly water-soluble, but not taken up by cells, like *Leishmania*. These cells are thus light-insensitive and remain fully viable, as indicated by their active motility when bathed in URO-containing milieu [5]. This changes dramatically for uroporphyrinogenic *Leishmania,* which are transgenically modified to express the 2^nd^ and 3^rd^ enzymes in the heme biosynthetic pathway, rendering them inducible with the product of the 1^st^ enzyme in this pathway, i.e. delta-aminolevulinate (ALA) for cytosolic accumulation of URO [5–7]. During ALA-induced uroporphyrinogenesis, these mutants cease flagellar motility abruptly when examined under dim light for microscopy as URO begins to form in the cytosol [5, 6]. Clearly, intracellular delivery of PS even in a minute amount is sufficient to sensitize cells to photo-inactivation.

**Fig. 1:**
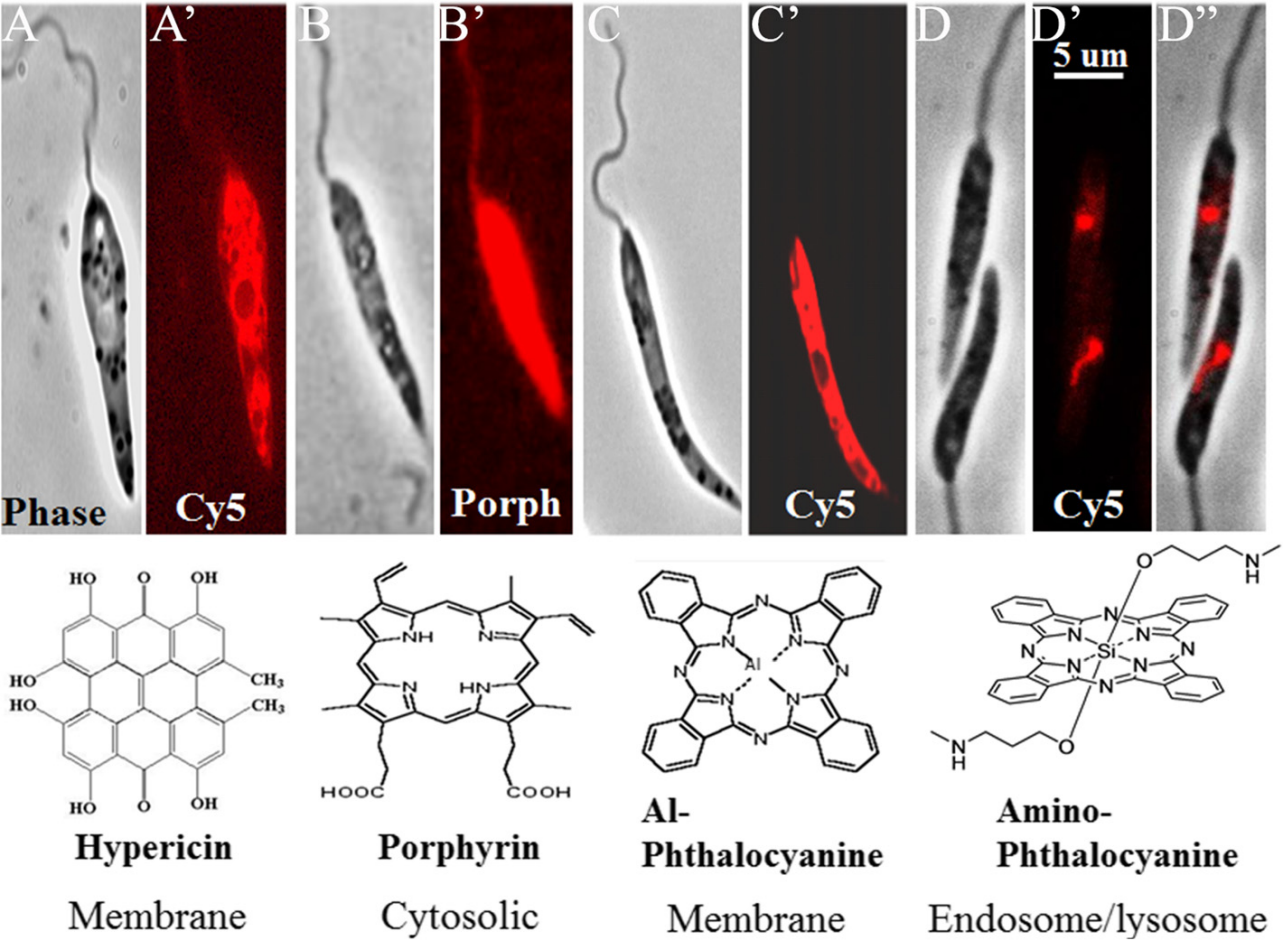
Photosensitization of *Leishmania* promastigotes with different photosensitizers. A-D, Phase contrast; A’-D”, Fluorescence images taken under Cy5 or porphyrin filters. Under each image are the name, structure and cellular localization of the photosensitizer used. Cells were exposed to each photosensitizer overnight in the dark and imaged under live conditions as previously described [6, 7, 10–12]

Cellular uptake of PS varies with their chemical structures. PC have been chemically modified to enhance such bioavailability. Modifications of their coordinating metals, side-chains and/or axial ligands increase cationicity for affinity to the negatively charged cell surface and solubility for persistence in the milieu [8, 9]. Figure 1 shows some PS, which are localized to different subcellular sites of *Leishmania*. ALA-induced cytosolic accumulation of URO was discussed earlier (Fig. 1B, B’). The hydrophobic/lipophilic hypericin (A, A’) and aluminum phthalocyanine (Al-PC) (C, C’) are taken up rapidly. These PS become associated immediately with and remain bound constantly to cellular membranes with undiminished fluorescence, but are transferrable from sensitized cells to the membranes of untreated cells [10]. How these phenomena are related to the expected turnover of cellular membranes is a question of interest for investigation. In contrast, the amino-PC [9] is endocytosed by *Leishmania* into their endosome-lysosome vacuolar system [11]. Other Si- or Zn-PC analogues [8] are either not taken up at all by *Leishmania* or are taken into the endosome-lysosome system or mitochondria [12].

Illumination of the PC-sensitized *Leishmania* with red light (~600 nm excitation wavelength) at low fluence (1–2 J cm^2^) generates enough ^1^O_2_ to inactivate them [11, 12]. The inactivated cells lose their flagellar motility and viability, but remain intact structurally for hours before disintegration. In many instances, *Leishmania* differ from mammalian cells in their response to different PS for PDT. Elucidation of these differential mechanisms is of interest for optimizing the utility of PS for targeted PDT.

### PDT in clinical use: PDT of cutaneous leishmaniasis (CL) and post-treatment immune clearance of infection

PDT is an accepted clinical regimen for treating solid tumors and skin diseases, and for removing diseased tissues [4]. PDT begins with PS-sensitization of the target tissues with a PS or an inducer of endogenous PS, i.e. ALA to transiently up-regulate cellular porphyrin biosynthesis. The sensitized target is then illuminated to generate ROS for its destruction. Clinical PDT is thus limited to superficial and localized targets, e.g. solid tumors accessible to PS-sensitization and to the subsequent photo-inactivation by illumination from an external light source. Targets several centimeters below the skin are still PDT-treatable by using PC excitable with deep-penetrating red light.

PDT has been explored for treating infectious diseases of the skin [1], including cutaneous leishmaniasis (CL). Various PS have been assessed for PDT of experimental and clinical CL using different light sources: LED, laser and sun light (see [11]). PDT has the potential to shorten the often protracted duration of simple CL before spontaneous healing. The ultimate cure of all infectious diseases is thought to depend on post-therapeutic immune clearance, since no drug is expected to reach all individual pathogens in any given infection, regardless of the dosages used and the frequency of applications. The “post-PDT immune clearance” of CL foretells the potential of photodynamic vaccination (PDV) for both immuno-prophylaxis and -therapy.

### Photodynamic vaccination (PDV)

Prophylactic vaccination is the best preventive measure against infectious diseases, especially zoonosis, which cannot be controlled readily because of its persistence in animal reservoirs (Cf. [13]). Here we describe PDV using PDT-inactivation of *Leishmania* for vaccination. The evolution of *Leishmania* for intra-antigen-presenting cells (APC) parasitism and their sensitivity to PDT via PS accumulation are exploited for developing strategies to optimize the efficacy and safety of PDV.

### PDT-inactivation of *Leishmania* for vaccination against leishmaniasis

#### Background

##### Lasting immunity after cure of leishmaniasis and “leishmanization”

Development of effective prophylactic vaccines for this disease has long been considered as feasible from the lasting or life-long immunity seen after spontaneous healing of simple CL and after chemotherapeutic cure of visceral leishmaniasis (VL) (Cf. [14]). Infection of healthy individuals with lesion-derived live parasites in a hidden place is the crudest form of vaccination for simple CL. This is known as “leishmanization” [15] and has been practiced for millennia in the endemic sites of the Middle East and Central Asia. The vaccinees develop lasting immunity after self-healing and are thus immune for life from the potentially facial disfiguring CL. The lasting immunity results from a T cell-mediated response to *Leishmania* naturally occurring vaccines, adjuvants and other immune-stimulating factors. The residence of *Leishmania* in APC makes these molecules readily available for processing and presentation, accounting very likely for the effective elicitation of cell-mediated immunity and the post-therapeutic immune clearance.

##### *Leishmania* vaccine availability, efficacy and safety

Vaccines are still under development for both human and canine leishmaniasis. “Leishmanization” is effective, but unacceptable unless accomplished without a full-blown leishmaniasis. The extensive literature on the use of cultured *Leishmania* as the vaccine sources has been exhaustively reviewed recently (see Supplemental Table 1 in [16]). Live vaccines using avirulent strains, drug-crippled parasites and genetically attenuated mutants have been examined in experimental animal models. Most extensively studied are inanimate vaccines from the following materials: (1) whole-cells of cultured *Leishmania* killed or inactivated by chemical or physical means, e.g. formalinization, heating/autoclaving and irradiation; (2) soluble or insoluble fractions of cultured *Leishmania* or their secretory products; and (3) recombinant products of immunologically active *Leishmania* antigens. Prophylactic efficacy has been shown for most of them against experimental leishmaniasis in animal models, but few have reached the stages of clinical trials. Of note from these trials are the findings that inanimate vaccines from categories (2) and (3) are safe and immunogenic [17–19], but are only partially effective at best against human and canine leishmaniasis. The only whole-cell vaccine examined in category (1) is ineffective, but proven safe, i.e. autoclaved promastigotes at a dose of ~200 ug (100–400 ug) ([20]; F. Modabber, personal communication). This dosage is equivalent to ~4 × 10^7^ promastigotes, comparable to the number used as leishmanin (up to 2 × 10^7^ promastigotes/dose in phenol or merthiolate) in Montenegro skin test for delayed type hypersensitivity (DTH) [21]. These chemically or physically inactivated promastigotes have been injected into several hundred thousands of people. The continuing use of leishmanin test for DTH attests to the safety of whole-cell *Leishmania* when inactivated appropriately.

Here we exploit PDT as a new modality of *Leishmania* inactivation for assessing the safety and efficacy of their use for vaccination.

### Three schemes of PDT-inactivated *Leishmaina* for vaccination

**Fig. 2:**
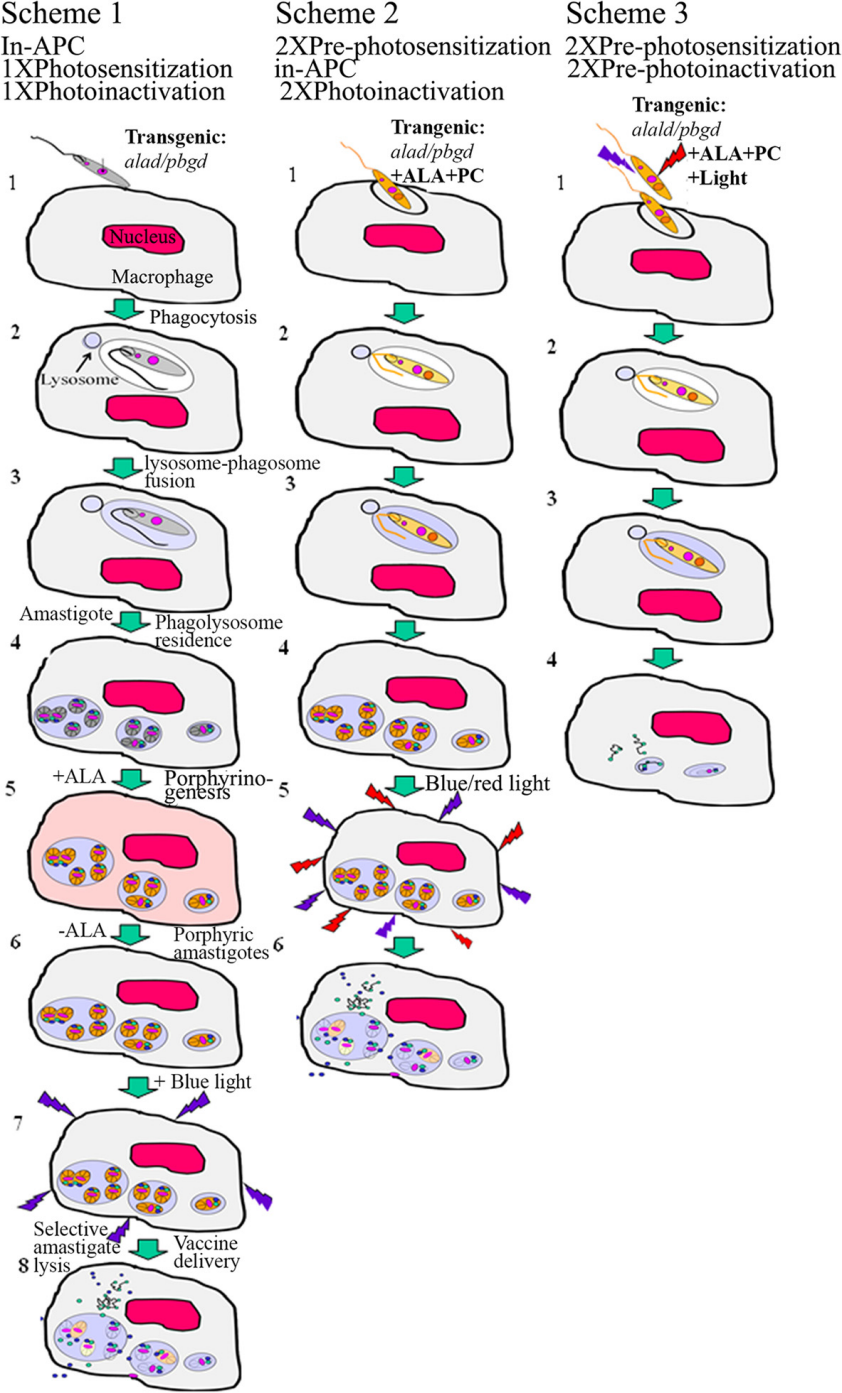
Diagramatic illustration depicting three different schemes of *Leishmania*-based photodynamic vaccination in vitro. Transgenic:*alad/pbgd*, Porphyrinogenic *Leishmania* transfected with two mammalian cDNAs encoding the 2^nd^ and 3^rd^ enzymes in heme biosynthetic pathway, rendering them susceptible to delta-aminolevulinate (ALA)-induced neogenesis of uroporphyrin (URO); PC, Si-phthalocyanine photosensitizer [6, 11, 12]; *Light*, Illumination; *Blue* and *red lightening symbols*, *Blue* (400–500 nm wavelength) and *red* (~600 nm wavelength) for excitation of URO and PC, respectively. Scheme 1: In-antigen presenting cell (APC) single PS-sensitization/photo-inactivation [22]. 1–2, Phagocytosis of porphyrinogenic, but untreated *Leishmania* by APC; 3, Fusion of *Leishmania*-containing phagosome with lysosome; 4, *Leishmania* differentiation into amastigotes and their replication in the phagolysosomes; 5, Exposure of the parasitized APC to ALA, resulting in porphyrinogenesis of both APC and phagolysosomal amastigotes; 6, Removal of ALA, resulting in disappearance of porphyrins from APC and persistence of URO in amastigotes; 7–8, Illumination of these APC resulting in selective lysis of URO-loaded amastigotes, releasing vaccines into phagolysosomes and cytosol. Scheme 2: Same as Scheme 1, except that porphyrinogenic *Leishmania* are doubly PS-sensitized with ALA and PC in the dark before use for infecting APC [35]. 1–4, as described for Scheme 1, except that the *Leishmania* are pre-loaded with URO and PC, hence no further ALA treatment; 5–6, Illumination of the infected cells with blue and red light to excite URO and PC, lysing amastigotes with singlet oxygen and other ROS generated for releasing vaccines in APC. Scheme 3: Same as Schemes 1–2, except that *Leishmania* are pre-PS-sensitized and pre-photo-inactivated before use for vaccine delivery to APC [12]. 1–4, Uptake of oxidatively photo-inactivated *Leishmania* by APC, lysosome-phagosome fusion and their lysis to release vaccines as described

The application of PDT in two steps (PS-sensitization followed by photo-inactivation) offers three different ways to inactivate *Leishmania* for vaccination, as depicted diagramatically in Fig. 2.

**Scheme 1** uses the uroporphyrinogenic *Leishmania* transfectants [5–7], which have the wildtype efficiency for entry into APC and differentiation/replication in their phagolysosomes [22] (**Events 1–4**). The 1^st^ PDT step is the addition of ALA to the infected APC, resulting in porphyrinogenesis of both the intra-phagolysosomal *Leishmania* transfectants and their host APC (**Event 5**). The latter become aporphyric shortly afterward, since they possess a complete heme biosynthetic pathway, thereby rapidly exhausting the excessive porphyrins produced; In contrast, the transgenic *Leishmania* produce URO, which persists and accumulates in their cytosol because of their deficient heme biosynthesis pathway, lacking the downstream URO-utilizing enzymes (**Event 6**). Light-exposure of these infected APC excites URO in the uroporphyric *Leishmania* for their selective inactivation (**Event 7**) and eventual lysis to release antigens into the phagolysosomes and cytosol of the viable host APC (**Event 8**).

**Scheme 2** is similar to Scheme 1, except that the uroporphyrinogenic *Leishmania* are doubly pre-PS-sensitized for the 1^st^ PDT step with ALA for URO accumulation in the cytosol and Si-PC for uptake into endosomes [11, 12]. These doubly PS-sensitized *Leishmania* infect APC in the dark, as described for Scheme 1 (**Events 1–4**). Subsequent light-exposure of these infected cells for the step 2 PDT produces the same outcome (**Event 6**), also as described for Scheme 1, except that the changes in the protocol reduce the events to 6 from 8 in Scheme 1.

**Scheme 3** is similar to Schemes 1–2, except that uroporphyrinogenic *Leishmania* are doubly PS-sensitized and photo-inactivated to complete both PDT steps as described for Scheme 2 before use for loading APC (**Event 1**). The changes of the protocol simplify the events to 4 from 6 to 8 for schemes 1–2. This scheme of APC-loading involves no replicative cycle of *Leishmania* in the host APC (**Events 2–4**).

#### Cell-mediated immunity depicted for PDT vaccination

##### Diagrammatic illustration

**Fig. 3:**
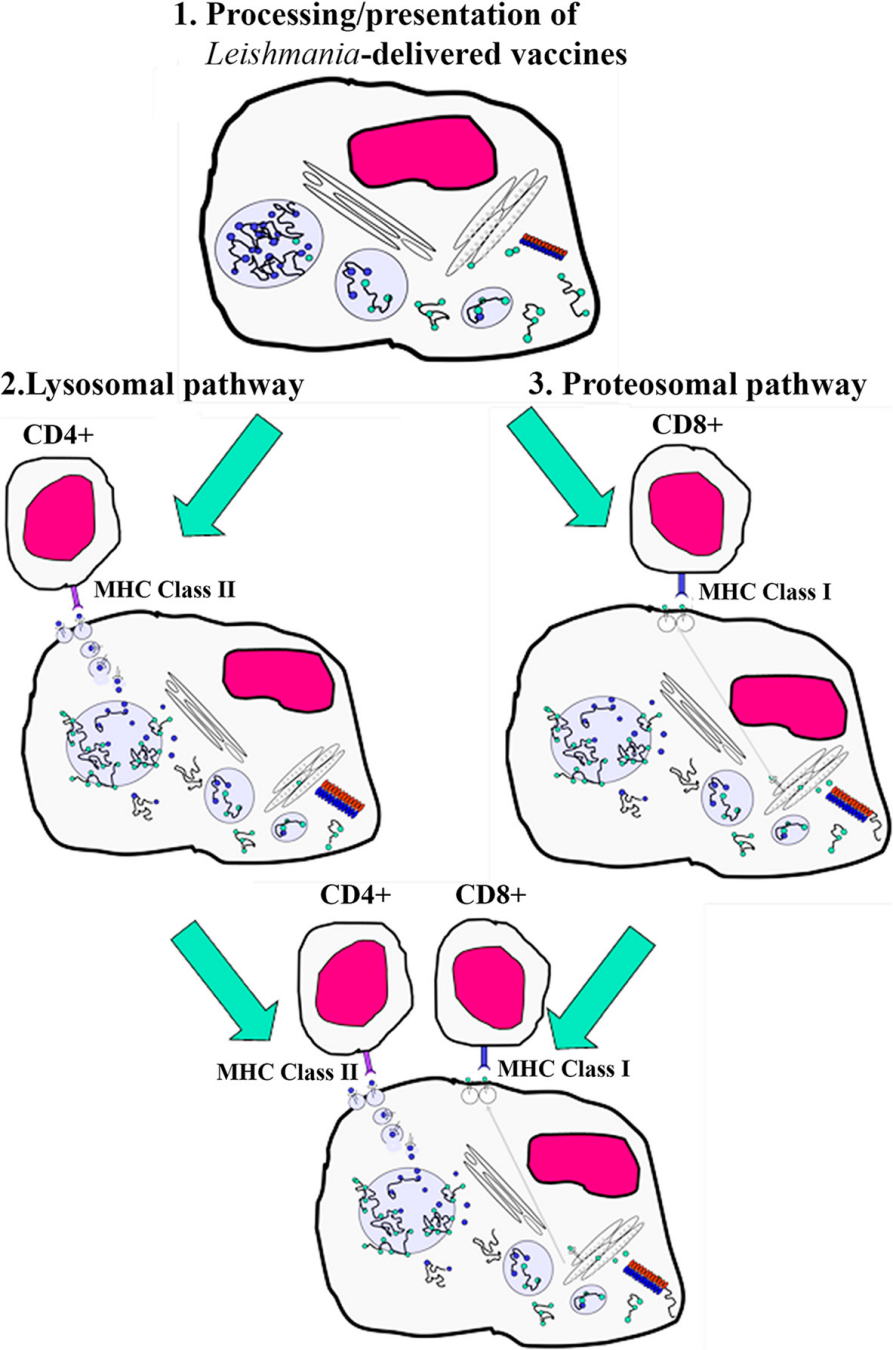
Diagrammatic depiction of processing and presentation of *Leishmania*-delivered vaccines by antigen-presenting cells. 1, *Leishmania*-released vaccines represented as dots and lines in phagolysosomes and cytosol and relevant organelles: RER, Golgi and proteasome stacks; 2, Antigen presentation by lysosomal pathway via MHC Class II for activation of CD4+ T cells; 3, Antigen presentation by proteosomal pathway via MHC Class I for activation of CD8+ T cells; *Bottom*, Combination of both pathways shown in 2 and 3

Figure 3 depicts the elicitation of cell-mediated immunity by all three PDV schemes based on experimental evidence described in the subsequent paragraphs. PDT selectively inactivates intracellular *Leishmania*, resulting in the eventual release of their contents into the viable host APC (**Event 1**). The materials released from photolysed *Leishmania* are expected to include antigenic vaccines and other putative immune stimulating factors, as depicted in the foregoing sections. Several pertinent issues are of interest to mention here. APC in schemes 1–2 remain unscathed and viable after PDT [22]. This is expected, since these host APC are not PS-sensitized at the time of illumination, and since the ^1^O_2_ produced is limited to the PS-sensitized *Leishmania*, as these ROS are too short-lived to cross multiple membranes to cause oxidative damage to the host APC. The endogenous anti-oxidants of APC are expected to protect themselves from other ROS generated secondarily from PDT. In addition, PDT may contribute positively to the APC functions in two ways: (1) Antigen processing by PDT-generated ^1^O_2_ and/or other ROS via oxidative modifications of the APC proteases involved and/or the *Leishmania*-released antigens as their substrates, e.g. ^1^O_2_ oxidation of their aromatic amino acid residues [23]; and (2) PDT-activation of ROS signal pathways favourable for the elicitation of immunity [24]. Clearly, the selective PDT-inactivation of intracellular *Leishmania* relieves their host APC of immunosuppression caused by the infection [22]. The subsequent processing of *Leishmania* vaccine antigens is predicted to follow the conventional lysosomal pathway (**Event 2**) and/or proteosomal pathway (**Event 3**) for co-presentation with MHC Class II and Class I molecules to activate CD4+ and CD8+ T cells, respectively (**Events 2–3**). The latter pathway is envisioned to proceed via cross presentation of *Leishmania* antigens, which are translocated from phagolysosomes to the cytosol. Other *Leishmaina*-derived factors may further participate in the step of co-stimulation (not shown).

##### Experimental evidence

The cell-mediated immunity depicted (Figs. 2 and 3) is based on the experimental outcome from the PDV schemes carried out in different experimental models, as briefly summarized below:

**Scheme 1** was applied to immunization of Syrian Golden hamsters, eliciting a Th1 response for prophylaxis against Indian kala-azar produced by challenges with virulent *Leishmania donovani* [25]. The vaccination produces lasting immunity, as shown by the analysis of hepatosplenomegaly, parasite loads and cytokine profiles. Significantly, the immunity is adoptively transferable by splenic T cells from immunized animals to naïve hamsters, indicating that the immunity is cell-mediated and requires no antigen stimulation from persistent parasites, if any, at least in the recipients.

**Fig. 4:**
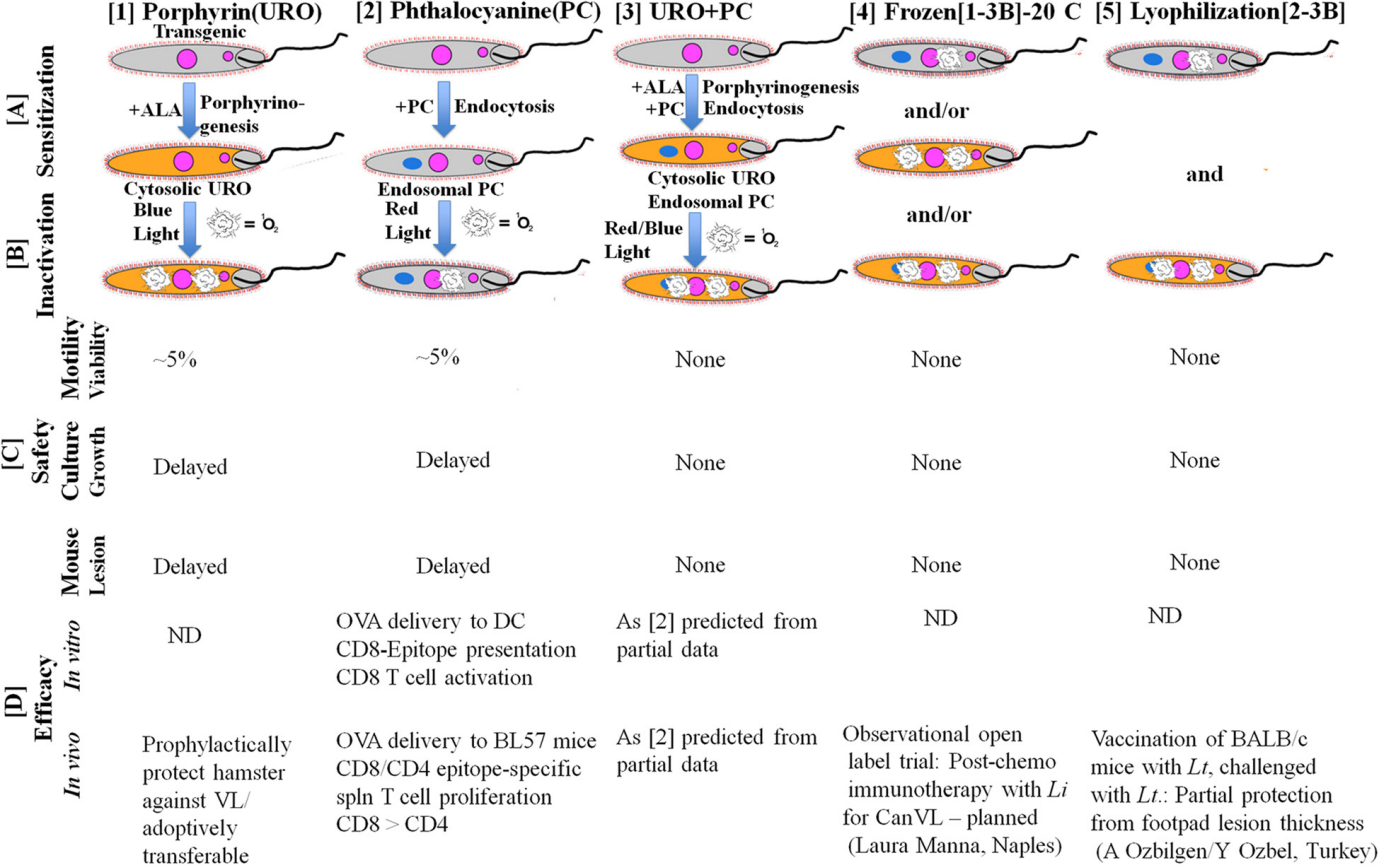
Safety and efficacy evaluations of PDT-inactivated *Leishmania* prepared under different conditions for immuno-prophylaxis and therapy. *Horizontal*: **[1]** and **[2]**, Single PS-sensitization with ALA (+ALA) for uroporphyrin (URO) or Si-PC-loading (PC) followed by single photo-inactivation with blue or red light illumination for generation of cytotoxic ^1^O_2_ (Symbol as shown), respectively [6, 7, 10–12]; **[3]**, Double PS-sensitization/double photo-inactivation using a combination of **[1]** and **[2]** conditions [35]; **[4**–**5]**, Singly or doubly PDT-inactivated *Leishmania* from **[1-3B]** stored frozen at −20 °C and lyophilized, respectively. See legend to Fig. 2 for other abbreviations used. *Vertical*: [A–B], PS-sensitization/photo-inactivation of *Leishmania* under the conditions as described in **[1–5]**. [C], Safety evaluations of the samples examined by microscopic observation for promastigote flagellar motility, for growth after inoculation into culture medium for 2 weeks, and lesion development after injection to BALB/c mouse ear dermis or tail base for ~60 days; [D], Efficacy evaluation in vitro and/or in vivo briefly summarized from published, on-going or planned studies. See text for **[1D]** and **[2D]** efficacy. Experimental-in-brief: see [25] for [1D in vivo]; see [12] for [2D in vitro]; [**2D in vivo**]: Groups of BL57 mice (~30 gm, 15/group) were immunized i.d. with 10^6^ photo-inactivated OVA transfectants/10 ul PBS/ear for 3 times 1-week apart. Control groups were simultaneously and similarly immunized with un-treated, PC-sensitized, light-exposed, freeze-thawed OVA-transfectants, and 1 ug OVA. Splenic cells were collected from four mice from each group 2-weeks after 1–3 immunization for in vitro activation with OVA CD4- and CD8-specific peptides for ~4 days. Proliferation of CFSE-labeled lymphocytes was assayed flow cytometrically, providing the results briefly described in the text; [**3D in vivo**]: Female BLAB/c mice (~30 g) were immunized exactly as described for [**2D in vivo**], except that doubly PS-sensitized *Leishmania* were used. Controls included 6 groups using untreated, single PS-sensitized, light-exposed samples. Day 3 after immunization, photo-inactivation of *Leishmania* was carried out *in situ* at ~5 J/cm^2^ using LumaCare LC-122 white light probe. Mice were each challenged at the tailbase with 10^7^ parasites. Lesion size was measured weekly in all groups. Experiments were terminated after ~10 weeks when parasite loads were determined by limiting dilution method. Preliminary results obtained were briefly described in the text. *Abbreviations*: CanVL, canine leishmaniasis; *Li, Leishmania infantum; Lt, Leishmania tropica*

**Scheme 2** was used for immunization of BALB/c mice against CL produced by challenges with *Leishmania amazonensis*. The observed prophylactic protection is significant, albeit incomplete, as indicated by comparing immunized mice *versus* the control groups. Immunization delayed the emergence of lesions for several weeks and significantly reduced the lesion size and their parasite loads by 10-fold *versus* the controls (Unpublished data. See legend to Fig. 4, Experimental-in-brief). The vaccination is considered effective, considering that BALB/c mice are known to bias toward Th2 with extreme levels of genetic susceptibility to cutaneous leishmaniasis.

**Scheme 3** PDV used PDT-inactivated *Leishmania,* which were transfected to express ovalbumin (OVA) as a marker antigen or surrogate vaccine [12]. The cell-mediated immune responses to OVA delivered by PDT-inactivated transfectants were examined in in vitro and in vivo mouse models. APC loaded with the PDT-inactivated *Leishmania* were shown to deliver OVA, which was effectively processed for MHC Class I presentation of its specific peptide for activation of CD8+ T cell line [12]. In the in vivo studies, BL57 mice were immunized three times, each with ~10^6^ PDT-inactivated OVA-*Leishmania*. Splenic T cells of these immunized mice were activated in response to CD4+ and CD8+ T cell-specific OVA peptides that increased proportionally with the number of immunizations (Unpublished data. See legend to Fig. 4, Experimental-in-brief). Most significantly, T cell activation is 6-fold higher with OVA delivered by PDT-inactivated *Leishmania* than that delivered by conventional means.

The safety of *Leishmania* PDT-inactivation for vaccination increases in the order of Schemes 1 to 3. *Leishmania* were singly and doubly PDT-inactivated for Scheme 1 and Schemes 2–3, respectively. They were completely inactivated by both PDT steps of PS-sensitization followed by double photo-inactivation before loading APC in Scheme 3 (see Fig. 4 and text for further discussion).

### PDT-inactivation of *Leishmania* for vaccine delivery against other infectious and malignant diseases

The utility of PDT-inactivated *Leishmania* for delivery of add-on vaccines against other diseases is feasible, as indicated by the favourable outcome of the immune responses seen in vitro *and* in vivo to OVA delivered by this means. The successful delivery of OVA is significant, considering its expression at minuscule amount against a background of *Leishmania* proteins in overwhelming quantity and diversity in ~10^6^ cells used for the delivery. This is taken to indicate that *Leishmania* creates no antigen-overload for vaccine delivery at least for OVA as a well-known T cell antigen.

*Leishmania* are naturally endowed with favourable attributes, making these parasites highly deployable as a universal vaccine carrier [22]. Many *Leishmania* species can be cultured safely as promastigotes in serum-free, chemically defined media [26] and scaled up for expansion [27]. The biosynthetic machineries of *Leishmania* are capable of high capacity transcription, translation and correct post-translational modification of foreign proteins. A number of efficient vectors are available for their abundant expression episomally or chromosomally as add-on vaccines in *Leishmania* - a favourable milieu of adjuvanticity and antigenicity conducive to elicit cell-mediated immunity.

Efficient delivery of add-on vaccines by *Leishmania* is due to their surface coat, consisting of unique lipid-saccharide-protein complexes [28]. In natural infection, they are known to protect *Leishmania* against the lytic humeral factors abundant in the animal body fluids and to target them to the phagolysosomes of APC. This mode of parasitism is further facilitated by the secretory products of *Leishmania*, e.g. nucleoside diphosphate kinase [29]. Full deployment of these molecular attributes by *Leishmania* is expected to protect the payload of add-on vaccines for homing to APC when using non-sensitized or PS-sensitized *Leishmania* for vaccine delivery according to Schemes 1–2 (Fig. 2). Notably, *Leishmania* PDT-inactivated according to Scheme 3 are no longer viable, but remain OVA-delivery competent. The integrity of their surface coat may account for this, since it is unaffected by the ^1^O_2_, which is generated in and limited to the cytosol of PDT-inactivated *Leishmania*.

Uroporphyrinogenic *Leishmania* are being evaluated for their ability to serve as a carrier of candidate vaccines for trials against other infectious and malignant diseases [30–33]. PDV with PDT-inactivated *Leishmania* transfectants will follow Schemes 1–3 (Fig. 2) to obtain safety and efficacy data. In vitro vaccination of DCs will be pursued, as described [33, 34]. This presents a new approach by using a eukaryotic vehicle for safe and effective vaccine delivery.

### Safety *versus* efficacy evaluation of five *Leishmania* PDT-inactivation formats

Figure 4 summaries the available data of *Leishmania*, which are PS-sensitized [A] and photo-inactivated [B] with or without additional treatments in different ways [1]–[5] for assessing their safety [C] and efficacy [D]. The safety is assessed after PDT inactivation of *Leishmania* by three different ways: microscopy for flagellar motility, cultivation for growth (2 weeks) and inoculation of mouse ear or tail base for lesion development (~2 months). Not all preparations were assessed by all criteria mentioned and the assessments for some samples are on-going or planned. The available results are briefly discussed below:

- **1.** Single PDT of *Leishmania* by ALA-induced uropoprhyrinogensis [1] or PC-loading [2] alone inactivated ~95 % of these cells, as determined by the criteria described [C]. Interestingly, PDV based on protocol [1] elicited adoptively transferable cell-mediated immunity and produced no visible pathology of the vaccination sites in hamster [25].
- **2.** Double-PDT of *Leishmania* with a combination of Protocols 1-2 [3] resulted in no viable cells, as assessed by all three criteria [C], indicative of a complete inactivation [35]. Immunization of BALB/c mice according to [3] is protective, although incomplete due to their inherent sensitivity to CL, as already discussed.

Products [4] and [5] prepared by freezing and lyophilization of PDT-inactivated *Leishmania* [1–3B], respectively, were undertaken to facilitate their storage and transport and to increase their safety at the expense of their efficacy. Although still on-going, lyophilized samples [5] appear to have some prophylactic activities against CL challenges after immunization of BALB/c mice.

From the available data, the double-PDT inactivation of *Leishmania* by method [3] provides the best vaccination format for use with optimal safety and efficacy. The other regimens are being optimized for further safety *versus* efficacy evaluation.

### Photodynamic insecticides (PDI)

#### Background

##### History

PDT to control insect pests was first mentioned in the early 1900’s (see [36]). From 1980’s to1990’s, The American Chemical Society published several symposium volumes on “Light-activated pesticides” [37–39]. Since then, follow-up publications have been limited and were summarized in the reviews [36, 40, 41]. Different dyes were used in experimental and/or field trials as PDI against various insects, mainly mosquito larvae and Mediterranean fruit flies. Industrial interests (PhotoDye International, Inc) included aerial spray of dye mixtures (xanthenes) or “SureDye®” (Red Dye #28 and Yellow Dye #8) (http://www.cdpr.ca.gov/docs/emon/pubs/ehapreps/suredye.htm) in attempt to control the latter pest. The work in the past decades showed some effectiveness of PDI, but this area of research has not gained attention.

##### Preamble

PDI has the potential as an effective measure to control disease-transmitting vectors and other harmful insects. Development of resistance by insect pests to insecticides is a recurrent scenario [42], calling attention to different approaches, like PDT, which is unlikely to elicit resistance. The potential of PDI to control different insect pests are briefly discussed below.

Phytophagous insects cause substantial losses in crops and livestock despite the use of genetically modified (GM) insect-resistant plants [43]. Phloem/xylem sap-feeding insects cause additional damage by transmitting plant diseases. These vectors are PDT-targetable, since they engorge voluminous plant saps amenable to PS-loading and are translucent to light for photo-inactivation. The use of ^1^O_2_-generating PS for PDT has the potential to discriminate these and other phytophagous insects for selective killing, sparing their photosynthetic and ^1^O_2_-resistant host plants.

Many animal biting insects feed on blood and transmit serious diseases, accounting for substantial morbidity and mortality of domestic animal and human populations worldwide. Application of PDI to control such insect vectors is highly desirable, e.g. *Anopheles* mosquitoes, which transmit malaria and *Aedes* spp., which transmit Chikungunya, Dengue and Zika fever, causing epidemics in the tropical/subtropical world today. The only new non-PDI approach to control these vectors is to release GM mosquitoes based on *Wolbachia*- or male-induced infertility [44, 45]. For PDT of female mosquitoes and other blood feeders (phototropic and day-light active species), PS is deliverable via the bloodstream of susceptible hosts or the use of suitable baits to sensitize the insects for sun light inactivation. The larval stages of all mosquitoes (and also black flies) are aquatic and thus are receptive to water-soluble PS for PDT [46, 47].

PS-sensitization of all insects is possible by direct spraying for their uptake via surface contact and/or systematically via the hosts, as used for the current insecticides. Direct incorporation of PS into the drinking and food sources of insects will deliver them into the digestive tracts for sensitization of cells therein. In either case, accessibility of PS-sensitized cells to light is necessary to generate cytotoxic ROS for target destruction. Nocturnal and darkness-loving insects are less amenable to PDT unless a light-emitter is provided with the PS for their excitation.

Summarized below are some observations from our preliminary studies of few insects on their uptake of selected PS and susceptibility to PDI.

### Screening of PS for their PDI against selected insects

**Fig. 5:**
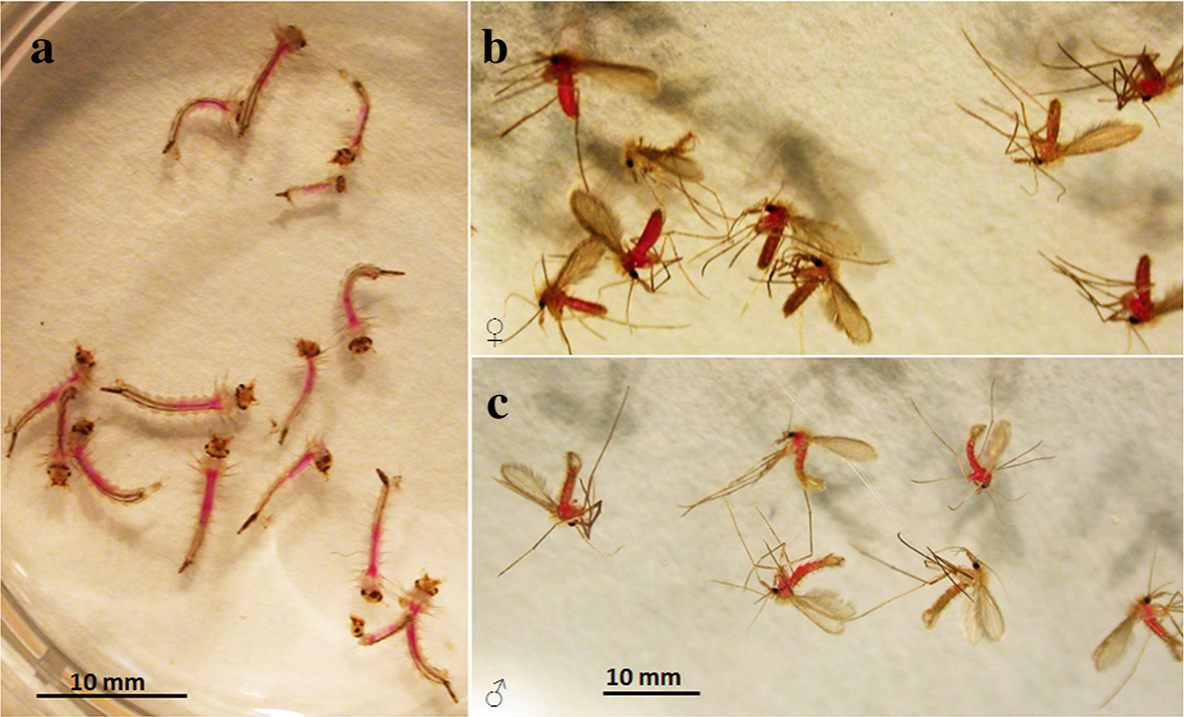
Uptake of rose bengal by selected insects and their photo-inactivation. **a** *Culex pipiens quinquefasciatus* 4th instar larvae exposed to rose Bengal (10 ug/ml) (~20 larvae/5 ml water/well in 6-well plate) in dark for ~24 hours followed by exposure to white light for ~6 h at ~2500 lux; **b**-**c** *Phlebotomus duboscqi* female (**b**) and male (**c**) adult flies (~20 flies/screened paper cup) fed with 5 % sucrose solution and 500 ul of 5 mg/ml rose bengal in a cotton ball for ~20 h in the dark followed by exposure to ~2500 lux of white light for 3 h. Duplicate samples were prepared and kept in the dark as controls. Rose bengal is taken up by the flies of both sexes. Phototoxicity is evident for the mosquito larvae, but inconclusive for the flies. The tests were done in Petr Volf’s lab

Exposure of the 4^th^ instar mosquito larvae (*Culex pipiens quinquefasciatus*) and adult sand flies (*Phlebotomus dubosqi*) [48] to rose bengal (RB) and cyanosine (CY) overnight resulted in the accumulation of these red dyes that are visible in the gut of the larvae (Fig. 5a) and of both female and male flies (Fig. 5b, c). Their uptake of the other PS examined is less clear, including aluminum-phthalocyanine (Al-PC), protoporphyrin IX (PROTO) and Nile blue sulfate (NB). Only RB- and CY-sensitized larvae lost their viability after light exposure based on their mobility (not shown). The sand fly response to the PDI is inconclusive due to a high mortality of the control group, pending further investigation. This is also true for PDI of the plant-sucking insects, e.g. aphids, suggestive of a need to use membrane-feeding techniques instead of using cut or potted plants [49, 50].

Our observations as described are preliminary, but represent the first study of PDI on sand flies, showing their uptake of PS used. The mosquito larvicidal activities of the PDI seen are consistent with the results of an early work (see [36]) and the reports using marigold alpha-terthienyl as the PS and different mosquito species [46, 47].

### Uptake of PS by mosquito cells in vitro

**Fig. 6:**
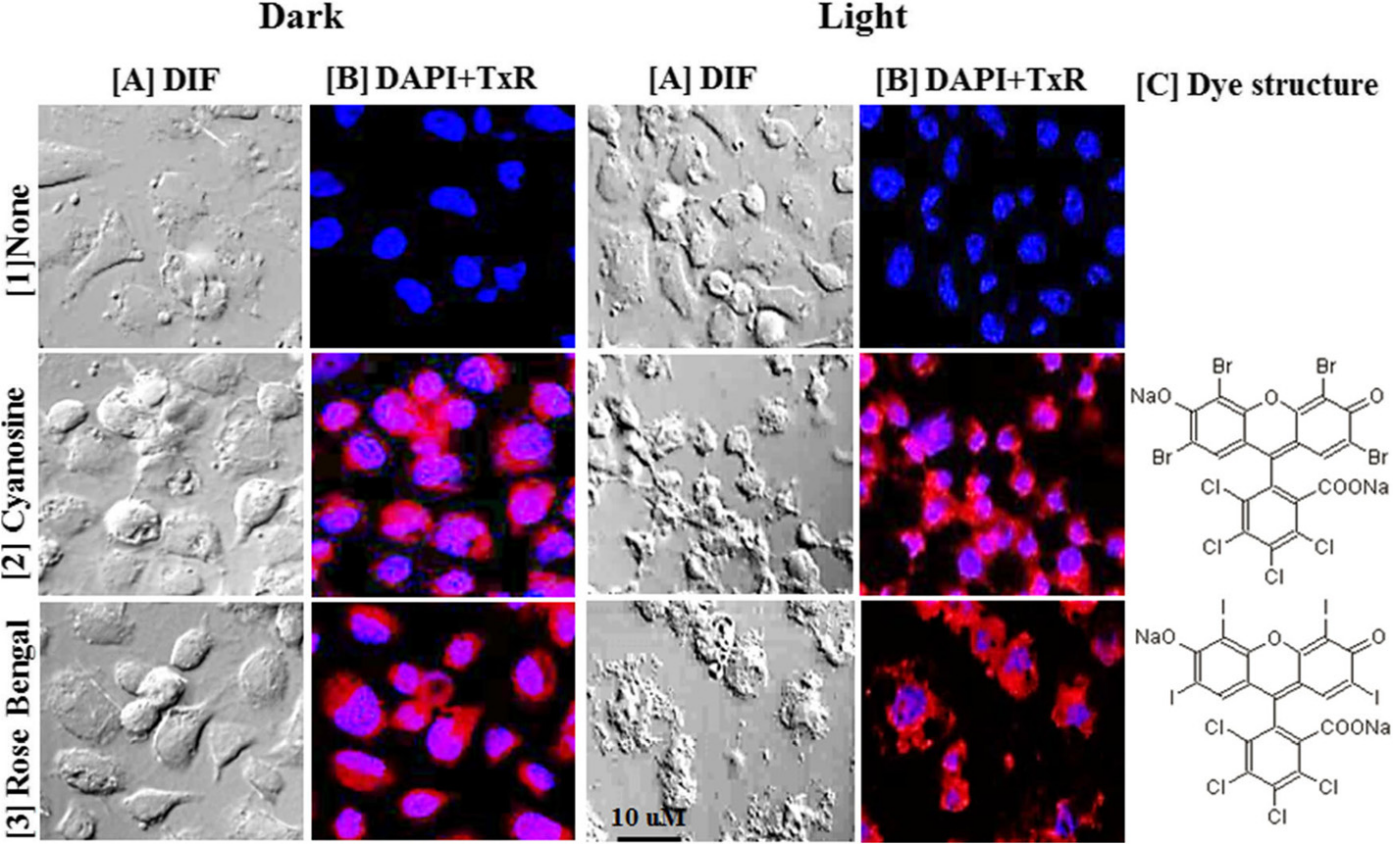
Uptake of cyanosine and rose bengal by mosquito cells of the C6/36 line and their photosensitivity in vitro. The insect cells were exposed to both dyes overnight and illuminated with white light under conditions similar to those described for mammalian and *Leishmania* cells [10–12, 22]. Images were captured first under differential interference (DIF) [A] and then under the filter sets for DAPI and Texas red [B]. [C] Chemical structure of cyanosione and rose Bengal. Uptake of both dyes by the cells after incubation in the dark overnight (*Dark*, A2-3, B2-3) and cellular disintegration after light exposure for 4 h (*Light*, A2-3, B2-3) in contrast to the untreated controls (Dark and Light, A1, B1). Work done by Shin-Hong Shiao

Since the uptake of PS by mammalian and *Leishmania* cells is a prerequisite for their sensitization for PDT, we have begun to assess this with insect cells, e.g. *Aedes albopictus* clone C6/36 (ATCC CRL-1660). Figure 6 shows the uptake of RB and CY by these mosquito cells, rendering them sensitive to photo-inactivation. Untreated cells ([1]-None) are adherent (1A-DIF) and non-fluorescent (1B DAPI + TxR), irrespective of illumination (1 Dark and Light). Cells exposed to CY [2] and RB [3] show cytoplasmic fluorescence (2B, 3B DAPI-txR), indicative of dye uptake. Sensitized cells remain adherent and intact (Dark, 2A, 3A-DIF), but become disintegrated after light-exposure (Light, 2A, 3A-DIF). These results are consistent with the larvicidal activities of RB and CY observed, providing a cellular basis for their PDT activities. Notably, the mosquito cells were not sensitized for PDT with the following PS: Al-PC [10], PC3-4 [11], NB and a porphyrin analogue [51]. Insect cells are thus similar to other cells in their requirement of PS uptake for susceptibility to PDT, but require different PS for PDI.

The preliminary data point to the feasibility of screening additional PS for PDT of cells from different insects, both harmful and beneficial, and from other life forms in their environments. Such in vitro screening of PS for activities has the potential to identify PDI, which discriminate harmful pests from beneficial insects and other friendly organisms for selective killing of the former. Of further interest is to elucidate the mechanisms of differential PS-uptake by cells of different origin, providing clues for designing PS with specificity for PDI targeting.

## Conclusions

PDT-inactivation of *Leishmania* offers the versatility and flexibility to balance safety *versus* efficacy for vaccination against leishmaniasis and as potential carriers of vaccines against other infectious and malignant diseases (PDV). The development of this new approach will benefit from governmental and public acceptance and support. The ingenuity of the new leadership [52] is needed for novel regulation that will ensure the safety of vaccines with no barrier to disrupt innovation. The advocacy groups also call attention to rectify the existing barriers between science and cures, e.g. fasterCures (http://www.fastercures.org/). Development of vaccines including PDV will further benefit from effective measures against the anti-vaccination movement [53].

PDI represents an alternative approach to control insect pests. It is still in its early infancy of development despite the idea first emerged almost 100 years ago. Many PS for PDI are innocuous compounds, which have long been used among our everyday household products. Their application as PDI is not expected to select for resistance in contrast to the chemical pesticides in current use. PDI has the potential to complement the GM approaches in the field of agriculture and medicine. It will be particularly suitable for development in places where the population is sensitive to GM organisms.

The lynchpin between PDV and PDI is the PS for light excitation to generate cytotoxic ROS. The expertise in medicinal chemistry is essential for synthesis and design of novel PS. This depends on the input of biologists to elucidate the mechanisms of their cellular/molecular activities. New PS need to be assessed by expert clinicians, veterinarians, entomologists, cancer researchers, microbiologists and immunologists, hence the consortium of collaborators enlisted.

## Abbreviations

ALA, delta-aminolevulinate; Al-PC, aluminum phthalycyanine; APC, antigen-presenting cells; CL, cutaneous leishmaniasis; CY, cyanosine; DIF, differential interference; DTH, delayed type hypersensitivity; GMO, genetically modified organisms; NB, Nile blue; OVA, ovalbumin; PC, phthalocyanines; PDI, photodynamic insecticide; PDT, photodynamic therapy; PDV, photodynamic vaccination; PROTO, protoporphyrin IX; PS, photosensitizer; RB, rose bengal; ROS, reactive oxygen species; URO, uroporphyrin I; VL, visceral leishmaniasis
